# Antibiotic Consumption and Deviation of Prescribed Daily Dose From the Defined Daily Dose in Critical Care Patients: A Point-Prevalence Study

**DOI:** 10.3389/fphar.2022.913568

**Published:** 2022-06-16

**Authors:** Patricia Helena Castro Nunes, Jessica Pronestino de Lima Moreira, Alessandra de Figueiredo Thompson, Thalita Lyrio da Silveira Machado, José Cerbino-Neto, Fernando Augusto Bozza

**Affiliations:** ^1^ D’Or Institute for Research and Education (IDOR), Rio de Janeiro, Brazil; ^2^ National Institute of Infectious Disease Evandro Chagas, Oswaldo Cruz Foundation (INI/FIOCRUZ), Rio de Janeiro, Brazil; ^3^ Faculty of Pharmacy, Fluminense Federal University, Niterói, Brazil; ^4^ Copa D’Or Hospital, Rio de Janeiro, Brazil

**Keywords:** intensive care unit, Anti-bacterial agents, drug utilization, antibiotic, consumption, defined daily dose, prescribed daily dose

## Abstract

**Background:** The consumption of antibiotics is one of the metrics used to evaluate the impact of antimicrobial stewardship programs (ASP). The aim of this study was to determine the prevalence of antibiotic consumption in Brazilian intensive care units (ICUs) and estimate the deviation of the prescribed daily dose (PDD) from the defined daily dose (DDD).

**Methods:** This is a multicenter, observational, point-prevalence study carried out in adult ICUs of 8 Brazilian hospitals from August 2019, to February 2020. We collected data on the patient’s demographic and clinical characteristics, antibiotic therapy, classification and site of infections. The DU90 (antibiotic accounting for 90% of the volume utilized) was calculated, and the antibiotics were classified by the Anatomical Therapeutic Chemical (ATC) Index and the World Health Organization (WHO) Access, Watch, Reserve (AWaRe) groups. For the most prevalent antibiotics, the deviation of PDD from DDD was determined.

**Results:** Three hundred thirty-two patients from 35 ICUs were analyzed. The prevalence of antibiotic use was 52.4%. The patients in use of antibiotics were predominantly over 60 years of age (81.6%) with pulmonary infections (45.8%). A predominance of empirical regimens was observed (62.6%) among antibiotic therapies. The highest frequencies of prescriptions observed were for piperacillin + tazobactam (16.1%), meropenem (13.3%), amoxicillin + clavulanate (7.2%), azithromycin (7.2%), and teicoplanin (6.1%). The watch (64.2%) and reserve (9.6%) categories of the AWaRe classification accounted for 73.8% of all antibiotics, and they were prescribed alone or in combinations. High variability of doses was observed for the most prescribed antibiotics, and large deviations of PDD from the DDD were observed for meropenem, teicoplanin, and tigecycline.

**Conclusions:** The high prevalence of antibiotic prescription was related to a predominance of empirical regimens and antibiotics belonging to the WHO Watch classification. High variability of doses and large deviations of PDD from DDD for meropenem, teicoplanin, and tigecycline was observed, suggesting that DDD may be insufficient to monitor the consumption of these antibiotics in the ICU population. The variability of doses found for the most prescribed antibiotics suggests the need for monitoring and intervention targets for antibiotic stewardship teams.

## Introduction

The consumption of antibiotics is an essential metric to estimate the impact of antimicrobial stewardship programs (ASP) on the rational use of antibiotics and the control of drug resistance. In critical care settings, ASP is especially challenged by frequent antibiotic use due to health care-associated infection, high prevalence of multiresistant bacteria, and systematic empirical therapy.

The defined daily dose (DDD) is a metric widely used to estimate and follow the consumption of drugs. However, it may not be ideal for evaluating antibiotics in intensive care units (ICUs) since the average daily dose for adults may not correspond to the mean daily dose prescribed (prescribed daily dose—PDD) to critical care patients. If the doses used are higher than the DDD (e.g., septic shock, nervous system infections) or lower (e.g., pediatric patients), the estimation of the number of days of therapy may not be accurate, making monitoring unreliable (World Health Organization, 2003; Polk et al., 2007; de With et al., 2009; Amadeo et al., 2010; Bitterman et al., 2016; Scheetz et al., 2016; Först et al., 2017). Alternative measures of consumption have been proposed (Stanic Benic et al., 2018). Among them, the direct measure of the number of days of therapy (DOT) has been recommended by the American Centers for Disease Control and Prevention (CDC National Healthcare Safety Network, 2022).

Recently, the WHO advocated the point prevalence methodology in low- and middle-income countries (LMICs) to estimate the prevalence of antibiotic use in hospitals (World Health Organization, 2018). In addition, few studies have analyzed the limitations of the DDD metric in the measurement of antibiotic consumption in adult ICUs. This study aimed to determine the point prevalence of antibiotic use in Brazilian adult ICUs and estimate the deviation of the mean PDD of antibiotics from their respective DDDs.

## Materials and Methods

### Study Design and Data Source

We performed a multicenter, observational, point-prevalence study prospectively conducted in a convenience sample of ICU patients of 8 hospitals in Rio de Janeiro, Brazil, part of the Rede D’Or network, the largest network of hospitals in Latin America. The study was coordinated by the Department of Critical Care of the D´Or Institute for Research and Education (IDOR), Rio de Janeiro, Brazil. Data were collected in a single day from August 5th, 2019, to February 20th, 2020. All adult inpatients in the selected ICUs hospitalized at or before 08:00 a.m. were considered eligible. We restricted the inclusion to ICUs registered in the Brazilian Research in Intensive Care Network (BRICNet, 2022) database and known to use the Epimed Monitor System^®^ (Epimed Solutions, 2021). The BRICNet is an independent research network for performing investigator-initiated multicenter studies in critical care in Brazil. Epimed Monitor System^®^ is a commercial cloud-based registry for quality improvement, performance evaluation, and benchmarking.

We analyzed the prescriptions of all patients hospitalized at the ICUs on the day of inclusion. A Research Electronic Data Capture (RedCap, 2022) form was developed to collect 1) ICU data: hospital name, ICU name, number of active beds, number of occupied beds (denominator); 2) clinical data: age, sex, weight, date and diagnosis of entry, diagnosis of infection; 3) information regarding patients with prescription of antibiotics (numerator): dose, dosage, type of therapy (prophylactic, empirical, definitive, other), route of administration, prescribed daily dose, creatinine clearance. We also used the Epimed Solutions^®^ program to obtain primary and secondary diagnoses (hospital admission and current admission to the ICU), origin, type of admission, Simplified Acute Physiology Score (SAPS 3, Metnitz et al., 2005; Moreno et al., 2005), Sequential and Organ Failure Assessment (SOFA, Vincent et al., 1996) scores on the day of admission to the ICU and data regarding comorbidities and ICU supportive use data (amines, mechanical ventilation, hemodialysis, urinary catheter, and central venous catheter). Attending physicians were directly consulted at the moment of filling Red Cap. The form was filled with references to each antibiotic prescribed. Thus, it was possible to identify the type of therapy (e.g., empirical, definitive, prophylactic), the site of infection, and the bacterium (in the cases of definitive therapy) for each antibiotic. As physicians were directly consulted, it was also possible to identify antibiotics used for other purposes such as immunomodulation and prokinetics. Nurses, the electronic medical records (EMR) of the participating hospitals, and other information sources available were also directly consulted when necessary.

### Study Definitions

In this study, antibiotic therapy was classified as prophylactic, empirical, definitive or “other”. Antibiotic prophylaxis refers to the use of antibiotic(s) to prevent an initial infection, the recurrence or reactivation of an infection, or the prevention of infection by the elimination of a colonizing organism (Enzler et al., 2011). Empirical therapy refers to the use of antibiotic(s) during the period prior to bacterial isolation and antibiotic susceptibility test results. Definitive therapy refers to the use of antibiotic(s) oriented by microbiology results (McGregor et al., 2007). “Other” refers to the use of antibiotic(s) as prokinetic(s) or immunomodulators. The infections were diagnosed by the specific site or bacterial agent. They were classified as community or health care-associated infections. A health care-associated infection (HCAI) was considered if the date of the event occurred on or after the 3rd calendar day of admission to an inpatient location where the day of admission was calendar Day 1 (CDC, 2019). The sites of infection were defined as follows: bloodstream, intraabdominal, nervous system, pulmonary, skin and soft tissues, urinary tract, non identified and “other”. We defined multidrug-resistant bacteria as those nonsusceptible to at least one agent in three or more antimicrobial categories, as described by Magiorakos et al., 2012.

We only analyzed antibiotics prescribed for systemic use. The exceptions were tobramycin for inhalation and vancomycin by oral/enteral inhalation, which were also included. One therapy with antibiotic(s) was considered a prescription of one or more antibiotics directed to one site of infection and/or to one bacterial agent. If the patient was receiving treatment for more than one site of infection and/or for a polybacterial infection and/or for another type of use (e.g., prophylactic, prokinetic, immunomodulator), each one was considered a different therapy.

The antibiotics were classified using the Anatomical Therapeutic Chemical (ATC) Classification System 2021 and WHO AWaRe Classification Database of Antibiotics 2021 for evaluation and monitoring. In the ATC classification system, the active substances are classified in a hierarchy with five different levels. The system has fourteen main anatomical/pharmacological groups or 1st levels. Each ATC main group is divided into 2nd levels, which could be either pharmacological or therapeutic groups. The 3rd and 4th levels are chemical, pharmacological, or therapeutic subgroups, and the 5th level is the chemical substance. The 2nd, 3rd, and 4th levels are often used to identify pharmacological subgroups that are considered more appropriate than therapeutic or chemical subgroups (World Health Organization, 2021a).

The AWaRe Classification is intended to be used as a tool for countries to better support antibiotic monitoring and stewardship activities. Antibiotics are classified into three groups: Access, Watch, and Reserve. The Access group includes antibiotics that have activity against a wide range of commonly encountered susceptible pathogens while also showing lower resistance potential than antibiotics in the other groups. The Watch group includes antibiotic classes with higher resistance potential and/or antibiotics at relatively high risk of selection for bacterial resistance. These medicines should be prioritized as critical targets of stewardship programs and monitoring. Last, the Reserve group includes antibiotics and antibiotic classes that should be reserved for the treatment of confirmed or suspected infections due to multidrug-resistant organisms. These medicines could be protected and prioritized as key targets of national and international stewardship programs involving monitoring and utilization reporting to preserve their effectiveness (World Health Organization, 2021b). In the analysis of the treatments containing antibiotic combinations, medicines containing beta-lactam associated with beta-lactamase inhibitors were not considered combinations.

### Data Processing and Statistical Analysis

Data analysis was performed using SPSS software. Absolute and relative frequencies and interquartile ranges (IQRs) of demographic and clinical characteristics of the studied individuals were described. The calculation of the total prevalence of the use of antibiotics and of the DU90 (antibiotics accounting for 90% of the volume utilized) was performed. For the most frequent antibiotics, the mean, median, minimum and maximum descriptive statistics of the PDD are presented. The absolute deviation of PDD from DDD was calculated using the formula DDD-PDD. The relative deviation was calculated using the formula [(DDD - PDD)/DDD)]*100. The one-sample *t* test was performed for the variable absolute deviation of PDD from DDD, using a test value equal to zero. A significance level of 5% was considered significant.

### Ethics

This study was conducted by the principles set out in the Declaration of Helsinki and the national and international ethical guidelines. The protocol was approved by the IDOR Institutional Review Board (register CAAE 03963418.8.0000.5249). The need for informed consent was waived. The principles of Good Clinical Practice (GCP) and other applicable regulations were used to guide procedures and considerations. Questionnaire collection was done with the RedCap system on tablets, and all data management was centralized in accordance with GCP and LGPD (Brazilian data protection law). Data integration and analysis were carried out on a secure server prepared for this purpose and conducted according to the analytical plan by the research team. The program was conducted with the clinical staff and those involved in the study ensured that participant’s privacy and confidentiality was maintained. Participants are not identified in any published reports of this study. All records kept confidential to the extent provided by international and local law. All evaluation forms, reports, study protocol, documentation, data and all other information generated will be held in strict confidence.

## Results

We analyzed the prescriptions of 332 adult patients from 35 ICUs of the 8 hospitals included in the study. Of these, 174 (52.4%) had a prescription for one or more antibiotics on the day of inclusion. The demographic and clinical characteristics of the 174 patients in antibiotic use(s) are summarized in Table 1. The patients were 54% female, and most were over 60 years of age (81.6%) and hospitalized due to clinical (nonsurgical) conditions (84.5%). The median length of stay (LoS) on the day of data collection was 8 days. The median SAPS 3 score was 53, and the median SOFA score was 1.5. Among the main reported comorbidities were hypertension (64.9%) and diabetes (35.1%). Eighty-nine of these patients (52%) had a diagnosis of sepsis. Among these septic patients, 50% had septic shock.

**TABLE 1:** Clinical and demographic characteristics of the critical care patients in use of antibiotic(s) (*n* = 174).

Characteristics	
Sex, N (%)	
Female	94 (54)
Age, median (IQR)	76 (64–86)
>60 years, N (%)	142 (81.6)
Admission type, N (%)	
Clinical	147 (84.5)
Elective surgery	17 (9.8)
Emergency surgery	10 (5.7)
Length of stay at the day of data collection, median (IQR)	8 (3,0–17,0)
Sepsis, N (%)	89 (52)
Septic shock N (%)	50 (28.7)
SOFA score^a^, median (IQR)	1.5 (0–4)
SAPS 3, median (IQR)	53 (44–65)
Creatinine Clearance, median (IQR)	67.5 (38,9–108,8)
Creatinine clearance <50 ml/min (Cockcroft and Gault), N (%)	55 (31.6)
Support in the ICU, N (%)	
Central venous catheter	102 (59)
Bladder catheter	95 (54.6)
Mechanical ventilation	67 (38.5)
Amines	58 (33.3)
Haemodialysis, N (%)	31 (17.8)
Comorbidities, N (%)	
High blood pressure	113 (64.9)
Diabetes	61 (35.1)
Chronic renal failure	32 (18.4)
Solid Tumour	32 (18.4)
Heart failure	15 (8.6)
Stroke	17 (9.8)
Immunosuppresion	16 (9.2)
COPD	13 (7.5)
Myocardial Infarction	11 (6.3)
Cirrhosis	6 (3.4)
Hepatic failure	2 (1.1)
Hematologic neoplasia	1 (0.6)

a N = 132 patients; COPD, Chronic obstructive pulmonary disease. IQR,Interquartile range.

We identified 201 antibiotic therapies. Most of them were empirical (62.6%), followed by definitive (23.4%) and prophylactic (7.5%). The number of infections was 171, and 52.6% were health care-associated infections. Of the 168 sites of infections observed in this study, the majority were pulmonary (45.8%) and intra-abdominal (15.4%) (Table 2 and Supplementary Table S1). Table 3 and Supplementary Table S2 describe the bacteria identified in the 47 infections with definitive use of antibiotics. These therapies were mainly directed to *Pseudomonas aeruginosa* (25.5%), which was identified mostly in respiratory (50%) and urinary infections (17%). Other frequent pathogens were *Staphylococcus aureus* (12.8%) and *Klebsiella* sp. (10.6%). Multidrug-resistant strains were also observed, such as *Pseudomonas aeruginosa* and *Klebsiella sp* ESBL-positive or carbapenem-resistant and methicillin-resistant *Staphylococcus aureus* (Table 3).

**TABLE 2:** Description of antibiotic therapy, classification and sites of infections of the critical care patients in use of antibiotic(s).

Antibiotic Therapy (*n* = 201)	N (%)
Empiric	126 (62.6)
Definitive	47 (23.4)
Prophylatic	15 (7.5)
Other	13 (6.5)
Classification of the infections (*n* = 171^a^)	
Comunity	81 (47.4)
Health care-associated	90 (52.6)
Site of infection (*n* = 168)	
Pulmonary	77 (45.8)
Intraabdominal	26 (15.4)
Urinary tract	21 (12.5)
Non identified	12 (7.1)
Skin and soft tissues	10 (6)
Bloodstream	6 (3.6)
Nervous system	3 (1.8)
Other**	13 (7.7)

a Two polybacterial infections; **Upper respiratory tract, arteriovenous fistula, osteomyelitis, mediastinum.

**TABLE 3:** Bacteria identified in the definitive treatments (*n* = 47) of the critical care patients in use of antibiotic(s).

Bacteria	N (%)	Multidrug Resistant N (%)
*Pseudomonas aeruginosa*	12 (25.5)	1 (2.1)
*Staphylococcus aureus*	6 (12.8)	2 (4.3)
*Klebsiella sp*	5 (10.6)	4 (8.5)
*Escherichia coli*	4 (8.5)	2 (4.3)
*Proteus sp*	4 (8.5)	0 (0)
Coagulase-negative Staphylococci	3 (6.4)	0 (0)
*Enterococcus* spp	3 (6.4)	1 (2.1)
Mycobacteria	2 (4.3)	0 (0)
*Stenotrophomonas maltophilia*	2 (4.3)	0 (0)
*Clostridium difficille*	2 (4.3)	0 (0)
*Enterobacter sp*	1 (2.1)	0 (0)
*Providencia Stuartii*	1 (2.1)	0 (0)
*Serratia sp*	1 (2.1)	0 (0)
Other	1 (2.1)	0 (0)

The total number of prescribed antibiotics was 279. Table 4 shows that the highest number of prescriptions was observed for piperacillin + tazobactam (16.1%), meropenem (13.3%), amoxicillin + clavulanate (7.2%), azithromycin (7.2%) and teicoplanin (6.1%). Erythromycin consumption (3.2%) was exclusively observed for its prokinetic indication. Table 4 also shows the DU90 for the antibiotics prescribed on the day of data collection and their AWare and ATC classification. In our study, we show that the main class of antibiotics prescribed to this population of critical care patients was from the watch group (64.2%) and that reserve antibiotics were also prescribed (9.6%) alone or in combination with antibiotics from the watch and access groups (Supplementary Tables S1, S2). We also show the prevalence of use of DU90 antibiotics in our population in Table 5. Another way to classify the data are through the therapeutic classes of the ATC classification system. In this case, the most frequent were beta-lactams, macrolides, glycopeptides, and aminoglycosides (Supplementary Tables S3–S6).

**TABLE 4:** ATC code, AWaRe classification, number of prescriptions (*n* = 279), relative frequencies and DU90 of the antibiotics prescribed to the critical care patients.

Antibiotic	ATC Code	AWaRe Classification	Number of Prescriptions N (%)	Cumulative Frequency (%)
Piperacillin + tazobactam	J01CR05	Watch	45 (16.1)	16.1
Meropenem	J01DH02	Watch	37 (13.3)	29.4
Amoxicillin + clavulanate	J01CR02	Access	20 (7.2)	36.6
Azithromycin	J01FA10	Watch	20 (7.2)	43.8
Teicoplanin	J01XA02	Watch	17 (6.1)	49.9
Amikacin	J01GB06	Access	12 (4.3)	54.2
Metronidazole	J01XD01 and PP01AB01^a^	Access	11 (3.9)	58.1
Ceftazidime	J01DD02	Watch	9 (3.2)	61.3
Erythromycin	J01FA01	Watch	9 (3.2)	64.5
Tigecycline	J01AA12	Reserve	8 (2.9)	67.4
Vancomycin	J01XA01 and A07AA09^a^	Watch	8 (2.9)	70.3
Cefuroxime	J01DC02	Watch	7 (2.5)	72.8
Linezolid	J01XX08	Reserve	7 (2.5)	75.3
Sulfamethoxazole + trimethoprim	J01EE01	Access	7 (2.5)	77.8
Ceftriaxone	J01DD04	Watch	6 (2.2)	80
Ciprofloxacin	J01MA02	Watch	6 (2.2)	82.2
Cefazolin	J01DB04	Access	6 (2.2)	84.4
Ampicillin + sulbactam	J01CR01	Access	5 (1.8)	86.2
Ertapenem	J01DH03	Watch	5 (1.8)	88.0
Polymyxin B	J01XB02	Reserve	5 (1.8)	89.8
Cefepime	J01DE01	Watch	3 (1.1)	90.9
Oxacillin	J01CF04	Access	3 (1.1)	92
Tobramycin	J01GB01	Watch	3 (1.1)	93.1

a When used for the treatment of Pseudomembranous Enterocolitis.

**TABLE 5:** ATC code, AWaRe classification and prevalence of use in the total population and in the population of critical care patients in use of antibiotics included in the DU90.

Antibiotic	ATC Code	AWaRe Classification	Prevalence (%) (*n* = 332)	Prevalence in the Patients Using Antibiotics (*n* = 174)
Piperacillin + tazobactam	J01CR05	Watch	13.5	25.8
Meropenem	J01DH02	Watch	11.1	21.2
Amoxicillin + clavulanate	J01CR02	Access	6.0	11.5
Azithromycin	J01FA10	Watch	6.0	11.5
Teicoplanin	J01XA02	Watch	5.1	9.8
Amikacin	J01GB06	Access	3.6	6.9
Metronidazole	J01XD01 and PP01AB01*	Access	3.3	6.3
Ceftazidime	J01DD02	Watch	2.7	5.2
Erythromycin	J01FA01	Watch	2.7	5.2
Tigecycline	J01AA12	Reserve	2.4	4.6
Vancomycin	J01XA01 and A07AA09*	Watch	2.4	4.6
Cefuroxime	J01DC02	Watch	2.1	4.0
Linezolid	J01XX08	Reserve	2.1	4.0
Sulfamethoxazole + trimethoprim	J01EE01	Access	2.1	4.0
Ceftriaxone	J01DD04	Watch	1.8	3.4
Ciprofloxacin	J01MA02	Watch	1.8	3.4
Cefazolin	J01DB04	Access	1.8	3.4
Ampicillin + sulbactam	J01CR01	Access	1.5	2.9
Ertapenem	J01DH03	Watch	1.5	2.9
Polymyxin B	J01XB02	Reserve	1.5	2.9
Cefepime	J01DE01	Watch	0.9	1.7
Oxacillin	J01CF04	Access	0.9	1.7
Tobramycin	J01GB01	Watch	0.9	1.7

The analysis of the deviation of the PDD from the DDD was performed for the most frequently prescribed antibiotics (Table 6). Larger percentage deviations with a *p* value <0.05 were observed for the antibiotics meropenem (−55.0%), teicoplanin (−79.4%) and tigecycline (−62.5%). The negative values of the deviations indicate that the mean PDDs were higher than their respective DDDs. It is also noteworthy that there is a high variability of prescribed doses for most of the antibiotics analyzed in Table 5, as we can see by analyzing their maximum and minimum values. These data point to the prescription of a varied repertoire of doses for this population of critical care patients.

**TABLE 6:** Descriptive statistics of the PDD and deviation of mean PDD from DDD of the most frequent antibiotics prescribed for the critical care patients.

Antibiotic	Number of Prescriptions	DDD (grams)	Mean PDD (grams)	Median PDD (grams)	IQR PDD	Minimum PDD (grams)	Maximum PDD (grams)	Absolute Deviation Mean PDD from DDD (%)	Relative Deviation Mean PDD from DDD (%)	SD Deviation of Relative Deviation	*p*-value of Deviation
Piperacillin + tazobactam	45	14.0	13.91	16.0	12.0–16.0	6.00	16.00	0.09	0.6	24.5	0.863
Meropenem	37	3.0	4.65	6.0	3.0–6.0	1.00	6.00	−1.65	−55.0	56.8	**<0.001**
Amoxicillin + clavulanate	20	3	2.85	3.0	3.0–3.0	1.00	3.00	0.15	5.0	16.3	0.186
Azithromycin	20	0.5	0.49	0.5	0.5–0.5	0.25	0.60	0.01	2.0	12.3	0.591
Teicoplanin	17	0.4	0.72	0.4	0.4–1.0	0.13	1.60	−0.32	−80.0	125.8	**0.020**
Amikacin	12	1.0	1.13	1.0	1.0–1.2	1.00	1.75	−0.13	−13.0	24.7	0.088
Metronidazole	11	1.5	1.39	1.5	1.5–1.5	0.75	1.50	0.11	7.3	17.3	0.176
Erythromycin	9	1.0	1.08	1.0	1.0–1.0	0.75	2.00	−0.08	−8.0	35.3	0.500
Tigecycline	8	0.1	0.16	0.2	0.1–0.2	0.10	0.20	−0.06	−60.0	51.8	**0.011**
Ceftazidime	8	4.0	4.25	4.5	2.25–6.0	2.00	6.00	−0.25	−6.3	47.7	0.722
Vancomycin	8	2.0	2.09	2.0	1.25–2.75	1.00	3.75	−0.09	−4.5	46.3	0.783

IQR, Interquartile range; SD, Standard deviation.

Significant deviation (*p* value < 0.05).

## Discussion

Point prevalence studies are being widely used to determine exposure to antimicrobials (European Centre for Disease Prevention And Control, 2016; Centers for Disease Control and Prevention, 2021; Global Point Prevalence Survey of Antimicrobial Consumption And Resistance; Global, 2022). The use of this method can be a strategy to overcome the lack of information on how antibiotics are prescribed and used at the patient level. Furthermore, in the vast majority of countries worldwide, continuous data collection on antibiotic prescribing is not possible due to the high workload and level of resources needed for regular monitoring (World Health Organization, 2018). Here, we used point prevalence methodology and found a high prevalence of antibiotic prescription (52.4%) to adult ICU patients. Most antibiotic therapies were empiric (62.6%), and the broad-spectrum antibiotics piperacillin + tazobactam and meropenem were the most prescribed. The watch and reserve categories of the AWaRe classification accounted for 73.8% of all antibiotics prescribed alone or in combinations. The mean PDDs of meropenem, teicoplanin, and tigecycline had significantly high deviations from their respective DDDs. These antibiotics were among the most used in these settings, suggesting that the use of DDD may be inadequate to analyze their consumption in critical care patients.

Disease in this study’s population seemed more severe than that described in the Brazilian ORCHESTRA study, which evaluated 59,693 patients from 78 national ICUs in 2013 (Soares et al., 2015). The median SAPS 3 score was 53 in our patients and 41 in the ORCHESTRA study. The percentages of patients with ICU support were also higher in our study: mechanical ventilation 38.5 vs 15.2%; vasopressors 33.3 *vs*. 12.8%; hemodialysis 17.8 *vs*. 2.8%. The high prevalence of antibiotic prescription found in this study (52.4%) is probably related to the severity of disease in the ICU population and the frequency of sepsis as a diagnosis. This prevalence is similar to those found for other ICUs in the world, even though in this case, the comparison is difficult because the studies are mainly performed for antimicrobials (not only antibiotics), and comprehensive clinical data of the patients have not been published. In the Brazilian Point Prevalence Study performed in 2017 at 18 hospitals, the authors found a prevalence of 60.3% for antimicrobial use in adult ICUs (Porto et al., 2020). In the Point Prevalence Survey conducted by Versporten et al. (2018) in 303 hospitals in 53 countries, the prevalence of antimicrobial (mainly antibiotics) use in ICUs ranged from 47.7% in West and Central Asia to 69.7% in Oceania. In Latin America, the prevalence was 55.1%. In Vietnam, a cross-sectional study performed in 2019 in 51 adult ICUs obtained a prevalence of 63.7% (Dat et al., 2021). In the United States of America (USA), the point prevalence survey conducted in 2015 by the Centers for Disease Control and Prevention obtained a prevalence of use of antimicrobials of 62.2% in ICUs (Magill et al., 2021).

The predominance of empirical treatments observed here is probably related to disease severity, the predominance of pulmonary infections, the high prevalence of sepsis, and the advanced age of patients. The high selective pressure favoring resistant bacteria and the local epidemiology might also be considered. Regarding the definitive therapies, we identified a predominance of the pathogens *Pseudomonas aeruginosa, Staphylococcus aureus* and *Klebsiella* sp. These bacteria are prevalent in the ICUs of Brazil, including resistant strains (Rossi, 2011; Subsecretaria de Vigilância em Saúde. Secretaria de Estado de Saúde, R. J. and Coordenação Estadual de Controle de Infecção Hospitalar, 2020). In 2017, the World Health Organization published the “Global priority list of antibiotic-resistant bacteria to guide research, discovery, and development of new antibiotics” (World Health Organization, 2017). Carbapenem-resistant *Pseudomonas aeruginosa* and *Klebsiella* sp. are considered critical by the World Health Organization and were identified in this work. *Staphylococcus aureus,* methicillin-resistant, and *Enterococcus sp* vancomycin-resistant are considered of high priority in this list and were also identified in this work. A finding that stands out is that we did not observe any infection caused by *Acinetobacter baumannii* in our study. This bacterium is prevalent in Brazilian ICUs (Rossi, 2011).

Previous studies on the consumption of antibiotics were conducted using various metrics, miscellaneous methodologies, and patient profiles. In addition, it should be noted that the current study was conducted before the COVID-19 pandemic was declared. Nevertheless, we consider that the results obtained for the most prescribed antibiotics present similarities to those obtained by recent studies performed in Brazilian ICUs (da Silva et al., 2021; Porto et al., 2020; Silva et al., 2021). In all cases, meropenem and piperacillin + tazobactam were among the most prescribed antibiotics. In addition, other antibiotics that are among our DU90 were found among the most prescribed antibiotics of these studies: vancomycin, cefepime (da Silva et al., 2021), ceftriaxone and vancomycin (Porto et al., 2020), tigecycline, polymyxin B, amoxicillin/clavulanate and amikacin (Silva et al., 2021). In the Point Prevalence Survey performed in 53 countries in 2015 (Versporten et al., 2018), the most prescribed antimicrobials were penicillins with a β-lactamase inhibitor, mainly piperacillin and amoxicillin.

On the other hand, studies from other LMICs observed that third-generation cephalosporins were the most consumed antibiotics (Axente et al., 2017; Alemkere et al., 2019; Zaha et al., 2019; Dat et al., 2021; Levy Hara et al., 2022). Considering the previous data, we can infer that despite using different methodologies, these studies point to the use of some frequently prescribed antibiotics for adult critical care patients who were found among our DU90. However, the comparison of antibiotic consumption data is inherently challenging. In addition to the varied methodologies and metrics that those authors used (e.g., prevalence, DDD or DOT/1000 patient-days), the differences found between the list of our most prescribed antibiotics and the lists obtained from other studies may be due to a combination of factors that can influence antibiotic use, such as the period of the study, case mix, patient comorbidities, specialty unit composition, differences in the local epidemiology of each of the ICUs analyzed, effective teams of antimicrobial stewardship, the sensitivity profiles of the local microorganisms (Markley et al., 2018) and the prevalence of septic patients. There is a direct impact of these features on the choice of empirical therapies and posterior de-escalation.

DDD is a universally accepted metric for drug utilization research (Hollingworth and Kairuz, 2021). Thus, the deviations between PDD and DDD found in this study may be due to the characteristics of ICU patients. In this study, we detected large deviations for the antibiotics meropenem, teicoplanin and tigecycline. We observed that they were prescribed in doses that were higher than their DDD. In addition, high dose variability was detected for these antibiotics and for others, such as piperacillin + tazobactam, ceftazidime, vancomycin and amikacin. These variabilities in PDD can be potential targets for antimicrobial stewardship teams and should be better studied. Our results seem to complement those of previous reports in the literature that show disparities between consumption data measured using the metrics DDD, PDD and DOT. Polk et al., 2007 analyzed antibiotic drugs administered to 1,074,174 adult patients discharged from 130 hospitals in the USA in 2005. They found that the measurement of aggregate hospital antibiotic use by DDD/1000 patient-days and DOT/1000 patient-days was discordant for many frequently used antibiotic drugs, such as ampicillin + sulbactam, cefepime, piperacillin + tazobactam and ceftriaxone. In 2006, de With et al., 2009 carried out a point prevalence study in a German hospital to quantify the differences between DDD, Recommended Daily Dose (RDD) and PDD. They found that large PDD/DDD discrepancies were noted for beta lactams and macrolides (clarithromycin). Alves & Martins (2013) performed a study in an adult ICU and found that the data of antimicrobial consumption in DDD/1000 patient-days obtained from the medical records differed from the same data obtained from the Hospital Pharmacy electronic dispensing reports. Relevant differences were found for antibiotics such as ampicillin + sulbactam, piperacillin + tazobactam and meropenem. More recently, Vallès et al. (2020) found that in critical care patients, global antimicrobial use was 36.7% higher when measured by DDDs than when measured by DOTs, and the PDD was greater than the DDD in more than 40% of the antibiotics. The authors also found major differences between DDD and DOT for 9 antibiotics, including meropenem.

In a systematic review followed by a RAND-modified Delphi consensus conducted by Stanic Benic et al., 2018, the authors propose a final set of 12 evidence-based and consensually validated quantity metrics for antibiotic use in the inpatient setting. Among them, there is a recommendation that antibiotic use should be preferably expressed in at least two metrics simultaneously. We believe that our data are compatible with this recommendation since the use of DDD may be insufficient to estimate the number of days of therapy (DOT) in the ICU population for the antibiotics meropenem, teicoplanin and tigecycline. The reasons for the high variability of doses of the most prescribed antibiotics may also represent an issue for investigation.

This work presents limitations that are intrinsic to its methodology. As a point prevalence study, incidence could not be reported, and long-term treatments may be overrepresented. Additionally, patients were not followed-up in time. Data from different hospitals were collected on different days, and we cannot exclude the possibility of seasonal differences.

In conclusion, this study reinforced the high prevalence of antibiotic use in a population of critically ill adults and provided additional evidence that the DDD metric presents limitations for monitoring antibiotic use in ICUs. More studies are necessary to determine the adequate metrics or combination of metrics. More accurate metrics may improve antimicrobial stewardship, benchmarking and future pooling of data in systematic reviews.

## Data Availability

The datasets presented in this article are not readily available because the dataset cannot be shared as it contains sensitive personal information from patients. Requests to access the datasets should be directed to FB, bozza.fernando@gmail.com.

## Abbreviations

ASP, Antimicrobial Stewardship Programs; ATC, Anatomical Therapeutic Chemical; AWaRe, Access, Watch, Reserve; BRICNet, Brazilian Research in Intensive Care Network; CDC, Centers for Disease Control and Prevention; COPD, Chronic obstructive pulmonary disease; DDD, Defined Daily Dose; DOT, Days of Therapy; DU90, antibiotic accounting for 90% of the volume utilized; HCAI, Healthcare-Associated Infection; ICU, Intensive Care Unit; IQR, Interquartile Range; LoS, Length-of-Stay; LMIC, Low-, Middle-Income Countries; MDR, Multidrug Resistance; MRSA, Methicillin-resistant *Staphylococcus aureus*; PDD, Prescribed Daily Dose; RedCap, Research Electronic Data Capture; RIPE, Rifampicin + Isoniazid + Pyrazinamide + Ethambutol; SAPS, Simplified Acute Physiology Score; SD, Standard deviation; SOFA, Sequential Organ Failure Assessment; USA, United States of America; VRE, Vancomycin-resistant Enterococci; WHO, World Health Organization.
