# Supplementary material for: Antibiotic Consumption and Deviation of Prescribed Daily Dose From the Defined Daily Dose in Critical Care Patients: A Point-Prevalence Study
Source: Front Pharmacol. 2022 Jun 16;13:913568. doi: 10.3389/fphar.2022.913568 (PMC9243753; doi:10.3389/fphar.2022.913568)
Supplement: Supplementary file 1 [file Table1.DOCX]

**SUPPLEMENT**

**Table of Contents**

Supplementary Table 1 2

Supplementary Table 2 7

Supplementary Table 3 13

Supplementary Table 4 14

Supplementary Table 5 15

Supplementary Table 6 16

**Supplementary table 1: Prescriptions of empirical therapy with antibiotics (n = 126) to critical care patients by site of infection, origin of infection, use of antibiotic combinations and AWaRe classification of the antibiotics.**

| **Site of infection**  **N (%)** | **Classification of the infection. N (%)** | **Antibiotic combination prescribed for**  **community pulmonary infections (n = 36)?**  **N (%)** | **Antibiotic therapy** | **AWaRe classification** | **Number of prescriptions for community pulmonary infections**  **(n = 36)**  **N (%)** |
| --- | --- | --- | --- | --- | --- |
| Pulmonary 64 (50.8) | Community 36 (28.6) | No  17 (47.2) | Piperacillin + tazobactam | Watch | 7 (19.4) |
|  |  |  | Amoxicillin + clavulanate | Access | 3 (8.3) |
|  |  |  | Meropenem | Watch | 2 (5.6) |
|  |  |  | Azithromycin | Watch | 1 (2.8)  Each one |
|  |  |  | Cefepime | Watch |  |
|  |  |  | Ertapenem | Watch |  |
|  |  |  | Levofloxacin | Watch |  |
|  |  |  | Sulfamethoxazole + trimethoprim | Access |  |
|  |  | Yes  19 (52.8) | Piperacillin + tazobactam + Azithromycin | Watch; Watch | 9 (25) |
|  |  |  | Amoxicillin + clavulanate + Azithromycin | Access; | 4 (11.1) |
|  |  |  | Amoxicillin + sulbactam + Azithromycin | Not classified; Watch | 2 (5.6) |
|  |  |  | Amikacin + Meropenem | Watch; Watch | 1 (2.8)  Each one |
|  |  |  | Cefuroxime + Azithromycin | Watch; Watch |  |
|  |  |  | Ceftazidime + Linezolid | Watch; Reserve |  |
|  |  |  | Piperacillin + tazobactam + Linezolid + Azithromycin | Watch; Reserve; Watch |  |
|  | **Classification of the infection N (%)** | **Antibiotic combination prescribed for**  **HCAI pulmonary infections (n = 28)?**  **N (%)** | **Antibiotic therapy** | **AWaRe classification** | **Number of prescriptions for HCAI pulmonary infections**  **(n = 28).**  **N (%)** |
|  | HCAI  28 (22.2) | No  11 (39.3) | Meropenem | Watch | 6 (21.4) |
|  |  |  | Piperacillin + tazobactam | Watch | 4 (14.3) |
|  |  |  | Clindamycin | Access | 1 (3.5) |
|  |  | Yes  16 (57.1) | Meropenem + Linezolid | Watch; Reserve | 3 (10.7)  Each one |
|  |  |  | Ceftazidime + Tigecycline | Watch; Reserve |  |
|  |  |  | Meropenem + Amikacin + Teicoplanin | Watch; Access; Watch | 1 (3.5)  Each one |
|  |  |  | Meropenem + Amikacin + Vancomycin | Watch; Access; Watch |  |
|  |  |  | Meropenem + Ciprofloxacin + Teicoplanin | Watch; Watch; Watch |  |
|  |  |  | Meropenem + Polymyxin B + Vancomycin | Watch; Reserve; Watch |  |
|  |  |  | Meropenem +Teicoplanin | Watch; Watch |  |
|  |  |  | Meropenem + Tobramycin (inalatory) | Watch; Watch |  |
|  |  |  | Meropenem + Vancomycin | Watch; Watch |  |
|  |  |  | Amikacin + Polymyxin B + Tigecycline | Access; Reserve; Reserve |  |
|  |  |  | Ertapenem + Linezolid | Watch; Reserve |  |
|  |  |  | Moxifloxacin + Tobramycin (inalatory) | Watch; Watch |  |

| **Site of infection, N (%)** | **Classification of the infection**  **N (%)** | **Antibiotic combination prescribed for community intraabdominal infections (n = 13)?**  **N (%)** | **Antibiotic therapy** | **AWaRe classification** | **Number of prescriptions for community intraabdominal infections (n = 13).**  **N (%)** |
| --- | --- | --- | --- | --- | --- |
| Intraabdominal 21 (16.7) | Comunity  13 (10.3) | No  9 (69.2) | Amoxicillin + clavulanate | Access | 2 (15.4) Each one |
|  |  |  | Ampicillin + sulbactam | Access |  |
|  |  |  | Ciprofloxacin | Watch |  |
|  |  |  | Ceftriaxone | Watch | 1 (7.7)  Each one |
|  |  |  | Meropenem | Watch |  |
|  |  |  | Metronidazole | Access |  |
|  |  | Yes  4 (30.8) | Ciprofloxacin + Metronidazole | Watch; Access | 3 (23.1) |
|  |  |  | Ceftriaxone + Metronidazole | Watch; Access | 1 (7.7) |
|  | **Classification of the infection**  **N (%)** | **Antibiotic combination prescribed for HCAI** **intraabdominal infections (n = 8)?**  **N (%)** | **Antibiotic therapy** | **AWaRe classification** | **Number of prescriptions for HCAI intraabdominal infections (n = 8)**  **N (%)** |
|  | HCAI  8 (6.3) | No  4 (50) | Piperacillin + tazobactam | Watch | 3 (37.5) |
|  |  |  | Meropenem | Watch | 1 (12.5) |
|  |  | Yes  4 (50) | Ceftazidime + Tigecycline | Watch; Reserve | 2 (25) |
|  |  |  | Ceftazidime + Teicoplanin | Watch; Watch | 1 (12.5)  Each one |
|  |  |  | Meropenem + Amikacin + Teicoplanin | Watch; Access; Watch |  |
| Urinary tract  8 (6.3) | **Classification of the infection**  **N (%)** | **Antibiotic combination prescribed for community urinary tract infections**  **(n = 4)?**  **N (%)** | **Antibiotic therapy** | **AWaRe classification** | **Number of prescriptions for community urinary tract infections**  **(n = 4)**  **N (%)** |
|  | Community  4 (3.2) | No  4 (100) | Piperacillin + tazobactam | Watch | 2 (50) |
|  |  |  | Cefuroxime | Watch | 1 (25)  Each one |
|  |  |  | Meropenem | Watch |  |
|  | **Classification of the infection N (%)** | **Antibiotic combination prescribed for HCAI**  **urinary tract infections**  **(n = 4)?**  **N (%)** | **Antibiotic therapy** | **AWaRe classification** | **Number of prescriptions for HCAI** **urinary tract infections (n = 4)**  **N (%)** |
|  | HCAI  4 (3.2) | No  4 (100) | Ceftriaxone | Watch | 1 (25)  Each one |
|  |  |  | Ertapenem | Watch |  |
|  |  |  | Piperacillin + tazobactam | Watch |  |
|  |  |  | Sulfamethoxazole + trimethoprim | Access |  |

| **Site of infection, N (%)** | **Classification of the infection**  **N (%)** | **Antibiotic combination prescribed for nonidentified community infections (n = 3)?**  **N (%)** | **Antibiotic therapy** | **AWaRe classification** | **Number of prescriptions for nonidentified community infections (n =3)**  **N (%)** |
| --- | --- | --- | --- | --- | --- |
| Nonidentified  10 (7.9) | Community  3 (2.4) | No  3 (100) | Ceftaroline-fosamil | Reserve | 1 (33.3) |
|  |  |  | Piperacillin + tazobactam | Watch | 2 (66.7) |
|  | **Classification of the infection N (%)** | **Antibiotic combination prescribed for nonidentified HCAI infections (n = 7)?**  **N (%)** | **Antibiotic therapy** | **AWaRe classification** | **Number of prescriptions for nonidentified HCAI infections (n = 7).**  **N (%)** |
|  | HCAI  7 (5.6) | No  3 (42.9) | Gentamicin | Access | 1 (14.3)  Each one |
|  |  |  | Meropenem | Watch |  |
|  |  |  | Piperacillin + tazobactam | Watch |  |
|  |  | Yes  4 (57.1) | Meropenem + Amikacin | Watch; Access | 1 (14.3)  Each one |
|  |  |  | Meropenem + Teicoplanin | Watch; Watch |  |
|  |  |  | Meropenem + Amikacin + Polymyxin B + Teicoplanin | Watch; Access; Reserve; Watch |  |
|  |  |  | Piperacillin + tazobactam + Teicoplanin | Watch; Watch |  |
| Skin and soft tissues  8 (6.3) | **Classification of the infection**  **N (%)** | **Antibiotic combination prescribed for community skin and soft tissues infections (n = 8)?**  **N (%)** | **Antibiotic therapy** | **AWaRe classification** | **Number of prescriptions for community skin and soft tissues infections**  **(n = 8)**  **N (%)** |
|  | Community  5 (4.0) | No  4 (50) | Amoxicillin + clavulanate | Access | 1 (12.5)  Each one |
|  |  |  | Cefepime | Watch |  |
|  |  |  | Ceftaroline-fosamil | Reserve |  |
|  |  |  | Cefuroxime | Watch |  |
|  |  | Yes  1 (12.5) | Piperacillin + tazobactam + Teicoplanin | Watch; Watch | 1 (12.5) |
|  | **Classification of the infection**  **N (%)** | **Antibiotic combination prescribed for HCAI skin and soft tissues infections (n = 3)?**  **N (%)** | **Antibiotic therapy** | **AWaRe classification** | **Number of prescriptions for HCAI skin and soft tissues infections (n = 3)**  **N (%)** |
|  | HCAI  3 (2.4) | Yes  3 (37.5) | Ampicillin + sulbactam + Clindamycin + Teicoplanin | Access; Access; Watch | 1 (12.5)  Each one |
|  |  |  | Meropenem + Teicoplanin | Watch; Watch |  |
|  |  |  | Piperacillin + tazobactam + Teicoplanin | Watch; Watch |  |

| **Site of infection, N (%)** | **Classification of the infection**  **N (%)** | **Antibiotic combination prescribed for HCAI** **bloodstream infections (n = 2)?**  **N (%)** | **Antibiotic therapy** | **AWaRe classification** | **Number of prescriptions for HCAI bloodstream infections infections (n = 2)**  **N (%)** |
| --- | --- | --- | --- | --- | --- |
| Bloodstream 2 (1.6) | HCAI  2 (1.6) | Yes  2 (100) | Piperacillin + tazobactam + Teicoplanin | Watch | 1 (50)  Each one |
|  |  |  | Sulfamethoxazole + trimethoprim (in combination with the antibiotics Meropenem + Linezolid + Ceftazidime + avibactam used as definitive therapy) | Access (definitive therapy: Watch; Reserve; Reserve) |  |
|  | **Classification of the infection**  **N (%)** | **Antibiotic combination prescribed for HCAI nervous system infections**  **(n = 1)?**  **N (%)** | **Antibiotic therapy** | **AWaRe classification** | **Number of prescriptions for HCAI nervous system infections (n = 1)**  **N (%)** |
| Nervous system  1 (0.8) | HCAI  1 (0.8) | Yes  1 (100) | Meropenem + Vancomycin | Watch; Watch | 1 (100) |
| Others  10 (7.9) | **Classification of the infection**  **N (%)** | **Antibiotic combination prescribed for other community infections (n = 7)?**  **N (%)** | **Antibiotic therapy** | **AWaRe classification** | **Number of Prescriptions for other community infections (n = 7)**  **N (%)** |
|  | Community  7 (5.6) | No  5 (71.4) | Amoxicillin + clavulanate | Access | 4 (57.1) |
|  |  |  | Piperacillin + tazobactam | Watch | 1 (14.3) |
|  |  | Yes  2 (28.6) | Cefepime + Teicoplanin | Watch; Watch | 1 (14.3)  Each one |
|  |  |  | Piperacillin + tazobactam + Teicoplanin | Watch; Watch |  |
|  | **Classification of the infection**  **N (%)** | **Antibiotic combination prescribed for other HCAI infections**  **(n = 3)?**  **N (%)** | **Antibiotic therapy** | **AWaRe classification** | **Number of prescriptions for other HCAI infections (n = 3)**  **N (%)** |
|  | HCAI  3 (2.3) | No  3 (100) | Meropenem | Watch | 1 (33.3)  Each one |
|  |  |  | Moxifloxacin | Watch |  |
|  |  |  | Piperacillin + tazobactam | Watch |  |

HCAI: Health care-associated infections.

S**upplementary table 2: Prescriptions of definitive therapy with antibiotics (n = 47) to critical care patients by site of infection, origin of infection, use of antibiotic combination and AWaRe classification of the antibiotics.**

| **Site of infection,**  **N (%)** | **Classification of the infection N (%)** | **Bacteria**  **N (MDR?)** | **Antibiotic combination prescribed for community pulmonary infections (n = 4)?**  **N (%)** | **Antibiotic therapy** | **AWaRe classification** | **Number of prescriptions for community pulmonary infections (n = 4).**  **N (%)** |
| --- | --- | --- | --- | --- | --- | --- |
| Pulmonary  13 (27.6) | Community 4 (8.5) | *Mycobacteria*  2 (No) | Yes  2 (50) | Levofloxacin + Ethambutol | Watch; Not classified | 1 (25)  Each one |
|  |  |  |  | RIPE | Not classified |  |
|  |  | *Pseudomonas aeruginosa*  1 (No) | No  1 (25) | Piperacillin + tazobactam | Watch | 1 (25) |
|  |  | Other*  1 (No) | No  1 (25) | Amoxicillin + clavulanate | Access | 1 (25) |
|  | **Classification of the infection N (%)** | **Bacteria**  **N (MDR?)** | **Antibiotic combination prescribed for HCAI pulmonary infections (n = 9)?**  **N (%)** | **Antibiotic therapy** | **AWaRe classification** | **Number of prescriptions for HCAI pulmonary infections (n = 9)**  **N (%)** |
|  | HCAI  9 (19.1) | *Pseudomonas aeruginosa*  4 (No) | No  2 (22.2) | Cefuroxime | Watch | 1 (11.1)  Each one |
|  |  |  |  | Piperacillin + tazobactam | Watch |  |
|  |  |  | Yes  2 (22.2) | Piperacillin + tazobactam + Tobramycin (inalatory) | Watch; Watch | 1 (11.1) |
|  |  |  |  | Amikacin + Polymyxin B + Tigecycline | Access; Reserve; Reserve | 1 (11.1) |
|  |  | *Pseudomonas aeruginosa*  1 (Yes, carbapenem-resistant) | No  1 (11.1) | Ceftazidime + avibactam | Reserve | 1 (11.1) |
|  |  | *Stenotrophomonas maltophilia*  2 (No) | No  2 (22.2) | Sulfamethoxazole + trimethoprim | Access | 2 (22.2) |
|  |  | *Serratia sp.*  1 (No) | No  1 (11.1) | Ceftriaxone | Watch | 1 (11.1) |
|  |  | *Staphylococcus aureus*  1 (No) | No  1 (11.1) | Oxacillin | Access | 1 (11.1) |

| **Site of infection,**  **N (%)** | **Classification of the infection N (%)** | **Bacteria**  **N (MDR?)** | **Antibiotic combination prescribed for HCAI intraabdominal infections (n = 6)?**  **N (%)** | **Antibiotic therapy** | **AWaRe classification** | **Number of prescriptions for HCAI ntraabdominal infections (n = 6)**  **N (%)** |
| --- | --- | --- | --- | --- | --- | --- |
| Intraabdominal 5 (10.6) | HCAI  6** (12.8) | *Clostridium difficille* 2 (No) | Yes  2 (33.3) | Metronidazole + Vancomycin (both by oral/enteral route) | Access; Watch | 2 (33.3) |
|  |  | Coagulase- negative *Staphylococci*  1 (No)** | No  1 (16.7) | Teicoplanin | Watch | 1 (16.7) |
|  |  | *Enterobacter sp* 1 (No)** | No  1 (16.7) | Piperacillin + tazobactam | Watch | 1 (16.7) |
|  |  | *Enterococcus spp* 2 (No) | No  2 (33.3) | Ampicillin | Access | 1 (16.7) Each one |
|  |  |  |  | Ampicillin + sulbactam | Access |  |

| **Site of infection,**  **N (%)** | **Classification of the infection N (%)** | **Bacteria**  **N (MDR?)** | **Antibiotic combination prescribed for community urinary tract infections (n = 8)?**  **N (%)** | **Antibiotic therapy** | **AWaRe classification** | **Number of prescriptions for community urinary tract infections (n = 8)**  **N (%)** |
| --- | --- | --- | --- | --- | --- | --- |
| Urinary tract  13 (27.6) | Community  8 (17) | *Escherichia coli* 1 (No) | No  1 (12.5) | Cefuroxime | Watch | 1 (12.5) |
|  |  | *Klebsiella sp* 1 (No) | No  1 (12.5) | Piperacillin + tazobactam | Watch | 1 (12.5) |
|  |  | *Klebsiella sp* 2 (Yes, ESBL) | No  2 (25) | Ertapenem | Watch | 1 (12.5)  Each one |
|  |  |  |  | Meropenem | Watch |  |
|  |  | *Proteus sp.* 3 (No) | No  3 (37.5) | Amoxicillin + clavulanate | Access | 1 (12.5) |
|  |  |  |  | Cefuroxime | Watch | 2 (25) |
|  |  | *Pseudomonas aeruginosa* 1 (No) | No  1 (12.5) | Ceftazidime | Watch | 1 (12.5) |
|  | **Classification of the infection N (%)** | **Bacteria**  **N (MDR?)** | **Antibiotic combination prescribed for HCAI urinary tract infections (n = 5)?**  **N (%)** | **Antibiotic therapy** | **AWaRe classification** | **Number of prescriptions for HCAI** **urinary tract infections (n = 5)**  **N (%)** |
|  | HCAI  5 (10.6) | *Escherichia coli* 1 (No) | No  1 (20) | Amoxicillin + clavulanate | Access | 1 (20) |
|  |  | *Escherichia coli* 2 (Yes, ESBL) | No  2 (40) | Ertapenem | Watch | 1 (20)  Each one |
|  |  |  |  | Meropenem | Watch |  |
|  |  | *Klebsiella sp* 1 (Yes, carbapenem-  resistant) | No  1 (20) | Amikacin | Access | 1 (20) |
|  |  | *Pseudomonas aeruginosa* 1 (No) | No  1 (20) | Meropenem | Watch | 1 (20) |

| **Site of infection,**  **N (%)** | **Classification of the infection N (%)** | **Bacteria**  **N (MDR?)** | **Antibiotic combination prescribed for nonidentified**  **community infections (n = 1)?**  **N (%)** | **Antibiotic**  **therapy** | **AWaRe classification** | **Number of prescriptions for nonidentified**  **community infections (n = 1)**  **N (%)** |
| --- | --- | --- | --- | --- | --- | --- |
| Nondentified  (2; 4.2) | Community 1 (2.1) | Coagulase- negative *Staphylococci*  1 (No) | No  1 (100) | Vancomycin | Watch | 1 (100) |
|  | **Classification of the infection N (%)** | **Bacteria**  **N (MDR?)** | **Antibiotic combination prescribed for nonidentified HCAI** **infections (n = 1)?**  **N (%)** | **Antibiotic therapy** | **AWaRe classification** | **Number of prescriptions for nonidentified**  **community infections (n = 1)**  **N (%)** |
|  | HCAI  1 (2.1) | *Pseudomonas aeruginosa*  1 (No) | Yes  1 (100) | Amikacin + Ceftazidime | Access; Watch | 1 (100) |
| Skin and soft tissues  2 (4.2) | **Classification of the infection N (%)** | **Bacteria**  **N (MDR?)** | **Antibiotic combination prescribed for HCAI** **skin and soft tissues infections (n = 2)?**  **N (%)** | **Antibiotic**  **therapy** | **AWaRe classification** | **Number of prescriptions for HCAI skin and soft tissues infections (n = 2)**  **N (%)** |
|  | HCAI  2 (4.2) | *Klebsiella sp* 1 (Yes, carbapenem-  resistant) | Yes  1 (50) | Amikacin + Polymyxin B | Access; Reserve | 1 (50) |
|  |  | *Staphylococcus aureus*  1 (No) | No  1 (50) | Oxacillin | Access | 1 (50) |

| **Site of infection,**  **N (%)** | **Classification of the infection N (%)** | **Bacteria**  **N (MDR?)** | **Antibiotic combination prescribed for HCAI**  **bloodstream infections (n = 6)?**  **N (%)** | **Antibiotic therapy** | **AWaRe classification** | **Number of prescriptions for HCAI** **bloodstream infections (n = 6)**  **N (%)** |
| --- | --- | --- | --- | --- | --- | --- |
| Bloodstream  4 (8.5) | HCAI**  6 (12.7) | Coagulase- negative *Staphylococci* 1 (No) | No  1 (16.7) | Daptomycin | Reserve | 1 (16.7) |
|  |  | *Enterococcus spp***  1 (Yes, VRE) | No  1 (16.7) | Linezolid | Reserve | 1 (16.7) |
|  |  | *Providencia Stuartii***  1 (No) | No  1 (16.7) | Meropenem | Watch | 1 (16.7) |
|  |  | *Pseudomonas aeruginosa*  1 (No) | No  1 (16.7) | Ceftazidime + avibactam | Reserve | 1 (16.7) |
|  |  | *Staphylococcus aureus*  2 (No) | No  2 (33.3) | Cefazolin | Access | 1 (16.7)  Each one |
|  |  |  |  | Oxacillin | Access |  |
| Nervous system  2 (4.2) | **Classification of the infection N (%)** | **Bacteria**  **N (MDR?)** | **Antibiotic combination prescribed for HCAI nervous system infections (n = 2)?**  **N (%)** | **Antibiotic therapy** | **AWaRe classification** | **Number of prescriptions for HCAI nervous system infections (n = 2)**  **N (%)** |
|  | HCAI  2 (4.2) | *Proteus sp.*  1 (No) | No  1 (50) | Ceftriaxone | Watch | 1 (50) |
|  |  | *Staphylococcus aureus*  1 (Yes, MRSA) | No  1 (50) | Vancomycin | Watch | 1 (50) |
| Others  3 (6.4) | **Classification of the infection N (%)** | **Bacteria**  **N (MDR?)** | **Antibiotic combination prescribed for other HCAI infections (n = 3)?**  **N (%)** | **Antibiotic therapy** | **AWaRe classification** | **Number of prescriptions for other** HCAI **infections (n = 3).**  **N (%)** |
|  | HCAI  3 (6.4) | *Pseudomonas aeruginosa*  1 (No) | No  1 (33.3) | Piperacillin + tazobactam | Watch | 1 (33.3) |
|  |  | *Pseudomonas aeruginosa* 1 (No) | No  1 (33.3) | Meropenem | Watch | 1 (33.3) |
|  |  | *Staphylococcus aureus*  1 (Yes, MRSA) | No  1 (33.3) | Daptomycin | Reserve | 1 (33.3) |

*Not reported. **Polybacterial infection. HCAI: Health care-associated infections; MRSA: Methicillin-resistant Staphylococcus aureus; MDR: Multidrug Resistance; RIPE: Rifampicin + Isoniazid + Pyrazinamide + Ethambutol; VRE: Vancomycin-resistant Enterococci.

**Supplementary table 3: ATC classification of third and fourth levels (WHO 2021a) of the antibiotics (n = 279) prescribed to critical care patients.**

| **ATC classification (third level)** | **N**  **(%)** | **Antibiotic l** | **N (%)** | **ATC classification**  **(fourth level)** | **N (%)** |
| --- | --- | --- | --- | --- | --- |
| J01D - Other beta-lactam antibacterials | 77 (27.6) | Cefazolin | 6 (2.2) | J01DB - First-generation cephalosporins | 6 (2.2) |
|  |  | Cefuroxime | 7 (2.5) | J01DC - Second-generation cephalosporins | 7 (2.5) |
|  |  | Ceftazidime + avibactam | 2 (0.7) | J01DD - Third-generation cephalosporins | 17 (6.1) |
|  |  | Ceftriaxone | 6 (2.2) |  |  |
|  |  | Ceftazidime | 9 (3.2) |  |  |
|  |  | Cefepime | 3 (1.1) | J01DE - Fourth-generation cephalosporins | 3 (1.1) |
|  |  | Ertapenem | 5 (1.8) | J01DH - Carbapenems | 42 (15.0) |
|  |  | Meropenem | 37 (13.3) |  |  |
|  |  | Ceftaroline-fosamil | 2 (0.7) | J01DI - Other cephalosporins and penems | 2 (0.7) |
| J01C - Beta-lactam antibacterials, penicillins | 76 (27.2) | Ampicillin | 1 (0.4) | J01CA Penicillins with extended spectrum | 1 (0.4) |
|  |  | Oxacillin | 3 (1.1) | J01CF - Beta-lactamase resistant penicillins | 3 (1.1) |
|  |  | Amoxicillin + sulbactam | 2 (0.7) | J01CR - Combinations of penicillins, incl. beta-lactamase inhibitors | 72 (25.8) |
|  |  | Ampicillin + sulbactam | 5 (1.8) | J01CR - Combinations of penicillins, incl. beta-lactamase inhibitors |  |
|  |  | Amoxicillin + clavulanate | 20 (7.2) | J01CR - Combinations of penicillins, incl. beta-lactamase inhibitors |  |
|  |  | Piperacillin + tazobactam | 45 (16.1) | J01CR - Combinations of penicillins, incl. beta-lactamase inhibitors |  |
| J01X - Other antibacterials | 52 (18.6) | Vancomycin | 8 (2.9) | J01XA - Glycopeptide antibacterials | 25 (9.0) |
|  |  | Teicoplanin | 17 (6.1) |  |  |
|  |  | Polymyxin B | 5 (1.8) | J01XB - Polymyxins | 5 (1.8) |
|  |  | Metronidazole | 11 (3.9) | J01XD - Imidazole derivatives | 11 (3.9) |
|  |  | Nitrofurantoin | 2 (0.7) | J01XE Nitrofuran derivatives | 2 (0.7) |
|  |  | Daptomycin | 2 (0.7) | J01XX - Other antibacterials | 9 (3.2) |
|  |  | Linezolid | 7 (2.5) |  |  |
| J01F - Macrolide, lincosamide and streptogramin | 31 (11.1) | Clarithromycin | 1 (0.4) | J01FA - Macrolides | 30 (10.8) |
|  |  | Erythromycin | 9 (3.2) |  |  |
|  |  | Azithromycin | 20 (7.2) |  |  |
|  |  | Clindamycin | 1 (0.4) | J01FF Lincosamides | 1 (0.4) |
| J01G - Aminoglycoside antibacterials | 16 (5.7) | Gentamicin | 1 (0.4) | J01GB - Other aminoglycosides | 16 (5.7) |
|  |  | Tobramycin | 3 (1.1) |  |  |
|  |  | Amikacin | 12 (4.3) |  |  |
| J01M - Quinolone antibacterials | 10 (3.6) | Levofloxacin | 2 (0.7) | J01MA - Fluoroquinolones | 10 (3.6) |
|  |  | Moxifloxacin | 2 (0.7) |  |  |
|  |  | Ciprofloxacin | 6 (2.2) |  |  |
| J01A - Tetracyclines | 8 (2.9) | Tigecycline | 8 (2.9) | J01AA - Tetracyclines | 8 (2.9) |
| J01E - Sulfonamides and trimethoprim | 7 (2.5) | Sulfamethoxazole + trimethoprim | 7 (2.5) | J01EE - Combinations of sulfonamides and trimethoprim, incl. derivatives | 7 (2.5) |
| J04A - Drugs for treatment of tuberculosis | 2 (0.7) | Ethambutol | 1 (0.4) | J04AK - Other drugs for treatment of tuberculosis | 1 (0.4) |
|  |  | RIPE | 1 (0.4) | J04AM - Combinations of drugs for treatment of tuberculosis | 1 (0.4) |

RIPE: Rifampicin + Isoniazid + Pyrazinamide + Ethambutol

**Supplementary table 4: ATC classification of third and fourth levels (WHO 2021a) of the antibiotics (n = 190) prescribed as empirical treatments to critical care patients.**

| **ATC classification (third level)** | **N (%)** | **Antibiotic** | **N (%)** | **ATC classification (fourth level)** | **N (%)** |
| --- | --- | --- | --- | --- | --- |
| J01C - Beta-lactam antibacterials, penicillins | 57 (30) | Piperacillin + tazobactam, | 38 (20) | J01CR - Combinations of penicillins, incl. beta-lactamase inhibitors | 57 (30) |
|  |  | Amoxicillin + clavulanate | 14 (7.4) |  |  |
|  |  | Ampicillin + sulbactam | 3 (1.6) |  |  |
|  |  | Amoxicilin + sulbactam | 2 (1.1) |  |  |
| J01D - Other beta-lactam antibacterials | 52 (27.4) | Meropenem | 32 (16.8) | J01DH - Carbapenems | 35 (18.4) |
|  |  | Ertapenem | 3 (1.6) |  |  |
|  |  | Ceftaroline-fosamil | 2 (1.1) | J01DI - Other cephalosporins and penems | 2 (1.1) |
|  |  | Cefepime | 3 (1.6) | J01DE - Fourth-generation cephalosporins | 3 (1.6) |
|  |  | Ceftazidime | 6 (3.2) | J01DD - Third-generation cephalosporins | 9 (4.7) |
|  |  | Ceftriaxone | 3 (1.6) |  |  |
|  |  | Cefuroxime | 3 (1.6) | J01DC - Second-generation cephalosporins | 3 (1.6) |
| J01X - Other antibacterials | 33 (17.4) | Teicoplanin | 15 (7.9) | J01XA - Glycopeptide antibacterials | 19 (10) |
|  |  | Vancomycin | 4 (2.1) |  |  |
|  |  | Linezolid | 6 (3.2) | J01XX - Other antibacterials | 6 (3.2) |
|  |  | Metronidazole | 5 (2.6) | J01XD - Imidazole derivatives | 5 (2.6) |
|  |  | Polymyxin B | 3 (1.6) | J01XB - Polymyxins | 3 (1.6) |
| J01F - Macrolide, lincosamide and streptogramin | 19 (10) | Azithromycin | 18 (9.5) | J01FA - Macrolides | 18 (9.5) |
|  |  | Clindamycin | 1 (0.5) | J01FF Lincosamides | 1 (0.5) |
| J01G - Aminoglycoside antibacterials | 10 (5.3) | Amikacin | 7 (3.7) | J01GB - Other aminoglycosides | 10 (5.3) |
|  |  | Gentamicin | 1 (0.5) |  |  |
|  |  | Tobramycin | 2 (1.1) |  |  |
| J01A - Tetracyclines | 7 (3.7) | Tigecycline | 7 (3.7) | J01AA - Tetracyclines | 7 (3.7) |
| J01E - Sulfonamides and trimethoprim | 3 (1.6) | Sulfamethoxazole + trimethoprim | 3 (1.6) | J01EE Combinations of sulfonamides and trimethoprim, incl. derivatives | 3 (1.6) |
| J01M - Quinolone antibacterials | 9 (4.7) | Ciprofloxacin | 6 (3.2) | J01MA - Fluoroquinolones | 9 (4.7) |
|  |  | Levofloxacin | 1 (0.5) |  |  |
|  |  | Moxifloxacin | 2 (1.1) |  |  |

**Supplementary table 5: ATC classification of third and fourth levels (WHO 2021a) of the antibiotics (n = 58) prescribed as definitive treatments to critical care patients.**

| **ATC classification (third level)** | **N (%)** | **Antibiotic** | **N (%)** | **ATC classification (fourth level)** | **N (%)** |
| --- | --- | --- | --- | --- | --- |
| J01C - Beta-lactam antibacterials, penicillins | 14 (24.1) | Piperacillin + tazobactam, | 6 (10.3) | J01CR - Combinations of penicillins, incl. beta-lactamase inhibitors | 10 (17.2) |
|  |  | Amoxicillin + clavulanate | 3 (5.2) |  |  |
|  |  | Ampicillin + sulbactam | 1 (1.7) |  |  |
|  |  | Ampicillin | 1 (1.7) | J01CA Penicillins with extended spectrum | 1 (1.7) |
|  |  | Oxacillin | 3 (5.2) | J01CF - Beta-lactamase resistant penicillins | 3 (5.2) |
| J01D - Other beta-lactam antibacterials | 18 (31) | Meropenem | 5 (8.6) | J01DH - Carbapenems | 7 (12.1) |
|  |  | Ertapenem | 2 (3.4) |  |  |
|  |  | Ceftazidime | 2 (3.4) | J01DD - Third-generation cephalosporins | 6 (10.3) |
|  |  | Ceftazidime + avibactam | 2 (3.4) |  |  |
|  |  | Ceftriaxone | 2 (3.4) |  |  |
|  |  | Cefuroxime | 4 (6.9) | J01DC - Second-generation cephalosporins | 4 (6.9) |
|  |  | Cefazolin | 1 (1.7) | J01DB - First-generation cephalosporins | 1 (1.7) |
| J01X - Other antibacterials | 12 (20.7) | Teicoplanin | 1 (1.7) | J01XA - Glycopeptide antibacterials | 5 (8.6) |
|  |  | Vancomycin | 4 (6.9) |  |  |
|  |  | Daptomycin | 2 (3.4) | J01XX - Other antibacterials | 3 (5.2) |
|  |  | Linezolid | 1 (1.7) |  |  |
|  |  | Metronidazole | 2 (3.4) | J01XD - Imidazole derivatives | 2 (3.4) |
|  |  | Polymyxin B | 2 (3.4) | J01XB - Polymyxins | 2 (3.4) |
| J01F - Macrolide, lincosamide and streptogramin | 1 (1.7) | Azithromycin | 1 (1.7) | J01FA - Macrolides | 1 (1.7) |
| J01G - Aminoglycoside antibacterials | 6 (10.3) | Amikacin | 5 (8.6) | J01GB - Other aminoglycosides | 6 (10.3) |
|  |  | Tobramycin | 1 (1.7) |  |  |
| J01A - Tetracyclines | 1 (1.7) | Tigecycline | 1 (1.7) | J01AA - Tetracyclines | 1 (1.7) |
| J01E - Sulfonamides and trimethoprim | 3 (5.2) | Sulfamethoxazole + trimethoprim | 3 (5.2) | J01EE Combinations of sulfonamides and trimethoprim, incl. derivatives | 3 (5.2) |
| J01M - Quinolone antibacterials | 1 (1.7) | Levofloxacin | 1 (1.7) | J01MA - Fluoroquinolones | 1 (1.7) |
| J04A - Drugs for treatment of tuberculosis | 2 (3.4) | Ethambutol | 1 (1.7) | J04AK - Other drugs for treatment of tuberculosis | 1 (1.7) |
|  |  | RIPE | 1 (1.7) | J04AM - Combinations of drugs for treatment of tuberculosis | 1 (1.7) |

RIPE: Rifampicin + Isoniazid + Pyrazinamide + Ethambutol

**Supplementary table 6: ATC classification of third and fourth levels (WHO 2021a) of prophylactic antibiotics (n = 16) prescribed to critical care patients.**

| **ATC classification (third level)** | **N (%)** | **Antibacterianos (frequência)** | **N (%)** | **ATC classification**  **(fourth level)** | **N (%)** |
| --- | --- | --- | --- | --- | --- |
| J01C - Beta-lactam antibacterials, penicillins | 5 (31.2) | Piperacillin + tazobactam, | 1 (6.2) | J01CR - Combinations of penicillins, incl. beta-lactamase inhibitors | 5 (31.2) |
|  |  | Amoxicillin + clavulanate | 3 (18.8) |  |  |
|  |  | Ampicillin + sulbactam | 1 (6.2) |  |  |
| J01D - Other beta-lactam antibacterials | 6 (37.5) | Ceftriaxone | 1 (6.2) | J01DD - Third-generation cephalosporins | 1 (6.2) |
|  |  | Cefazolin | 5 (31.2) | J01DB - First-generation cephalosporins | 5 (31.2) |
| J01X - Other antibacterials | 4 (25) | Metronidazole | 2 (12.5) | J01XD - Imidazole derivatives | 2 (12.5) |
|  |  | Nitrofurantoin | 2 (12.5) | J01XE - Nitrofuran derivatives | 2 (12.5) |
| J01E - Sulfonamides and trimethoprim | 1 (6.2) | Sulfamethoxazole + trimethoprim | 1 (6.2) | J01EE Combinations of sulfonamides and trimethoprim, incl. derivatives | 1 (6.2) |
